# Motivation and training needs of prison healthcare professionals: findings from a qualitative study

**DOI:** 10.1186/s40359-023-01076-8

**Published:** 2023-05-20

**Authors:** Benjamin Jeker, David Shaw, Nicolas Lagnaux, Tenzin Wangmo, Bernice S. Elger

**Affiliations:** 1 Medizinische Fakultät, University of Basel, Basel, Switzerland; 2 Institute for Biomedical Ethics, University of Basel, Basel, Switzerland; 3 Kantonsspital Graubünden, Chur, Switzerland; 4 Center for Legal Medicine, University of Geneva, Geneva, Switzerland; 5 Care and Public Health Institute, Maastricht University, Maastricht, The Netherlands

**Keywords:** Prison, Healthcare, Incarcerated patients, Training needs, Motivation, Prison medicine

## Abstract

Health care in prison is a challenging task. The conditions of imprisonment create distinct difficulties for those providing health care in this setting. These particular circumstances have led to a shortage of quality professionals, working for the health of imprisoned people. The aim of this study is to elaborate reasons for healthcare professionals to work in a prison environment. The main research question is: why do healthcare workers choose to work in prisons? Furthermore, our study identifies training needs in various fields. Interview data that comes from a national project carried out in Switzerland and three other relatively wealthy countries were analyzed using content analysis. One-on-one, semi structured interviews were designed and conducted with professionals working in prison context. A total of 105 interviews were carried out and for this work 83 of them were analyzed and coded into themes responding to the study aim. Most participants chose to work in prison either because of practical reasons, as many reported various forms of contact with the studied prison environment at a younger age, or because of intrinsic reasons, including among others, having the wish to change the system of healthcare in prisons. Even though the education of the participants varied greatly, a lack of specialist training was expressed by many health care professions as an important factor. This study points out the need for more specific training programs for healthcare workers in prison and provides suggestions to ameliorate the recruitment and education for future prison health care workers.

## Introduction

Providing health care in prison is challenging. The conditions of imprisonment create distinct difficulties for those providing health care in this setting. Due to the coercive environment and the frequent lack of resources, it is often difficult to respect the principle of equivalence of care [1–3]. This principle notes that patients in prison should receive care equivalent to that received by patients in the community [4], including treatments currently accessible only through research [5]. However, health care professionals trained in hospitals may not be appropriately prepared for working in prison [6–8]. This is because in the prison context, these professionals face not only medical problems, but also distinct social and legal issues. Therefore, in order to ensure the principle of equivalence, health care professionals working in prisons must also have necessary training and competency. Some countries have implemented the principle of equivalence by attaching prison health care to the health care service nationally to ensure its independence from the justice system [1]. In many other European countries, including many cantons in Switzerland, prison health care remains under the authority of the justice system [1–3]. This is contrary to international soft law which requires independence of medical care from the justice system [9]. Even when the provision of equivalent care is guaranteed, there are other barriers preventing health care professionals from working in prison, such as hesitation to work in an environment perceived as dangerous or hostile, and possible stigmatization from peers for working in prison. These issues become even more challenging when healthcare workers are faced with an ageing prison population, as older incarcerated people face additional challenges in accessing care as well as specific healthcare issues [10–18]. To our knowledge, there is no previous study on the topic of training for health care professionals and their motivation to work in prison. The present work attempts to fill this gap by identifying why and how health care professionals come to work with patients in prisons and which difficulties they face in seeking to provide good care in prison.

## Methods

This qualitative study is part of a larger national research project on the health of older persons in detention (‘Agequake in prisons—first part and second part’). Data for this paper comes from the overall project. The first part was carried out in Switzerland and two other wealthy countries [10] between 2012 and 2013. The second part was conducted from 2017 to 2019 in Switzerland and Canada [19]. The first part of the Agequake project focused on somatic health and social care provided to older persons in prisons as well as ethical and practical challenges that professionals working in this field experienced. In the second part, the project was extended with more data concerning the mental health of older people living in detention. Therefore, mental health care professionals were interviewed. The aim of including other countries was to compare different practices in other countries that have public health care systems and social health insurance.

In the Agequake project, an older incarcerated person was defined as any imprisoned person who was 50 years and older [13, 14] in light of their accelerated ageing [15, 16]. The participants' names and individual country identities (in two cases) are not provided to protect their anonymity, especially in the limited community of people working in prison.

For recruitment of the first part of the study, experts were found using purposive and convenience sampling. First, study collaborators recommended experts for the study (see [10] for details). Second, contact persons in the healthcare service or in the prison administration were asked to take part in the interviews or to give names of other possible interviewees in various prison settings. Additional experts were identified through their publications. They were ensured that it would not be possible to recognise neither a particular prison, nor a case, nor a situation, and that their anonymity would be preserved. To extend the research, in the second part further interviews with mental health professionals were conducted also using purposive sampling. First, participants were contacted by email or phone, and informed about the study. The inclusion and exclusion criteria of the two study parts differed; they are presented in the following Table 1.

**Table 1:** Participants inclusion and exclusion criteria

*Inclusion criteria*	
Agequake I	(a) Health care professional or researchers working in prison (b) Members of prison administration and policymakers (c) Members from relevant international and national NGOs
Agequake II	(a) Mental health professional (b) Substantial work experience in correctional context (minimum of 10 years work experience) (c) Work experience in either the German or French speaking language region of Switzerland
*Exclusion criteria*	
First and second part	(a) Italian speaking language region of Switzerland (that region is very small with only one correctional institution and interviewees would thus be easily identifiable) (b) Health professional not directly involved in the care of mentally ill patients

**Fig. 1:**
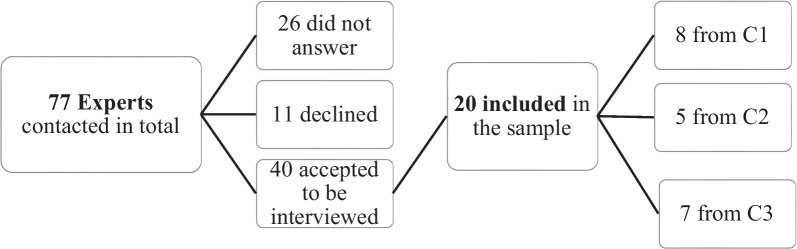
Participants recruitment and inclusion of Agequake part 1

Participants were interviewed personally by the researchers. At the scheduled time and place of the personal interview, the researchers explained again the purpose of the study, specified that all data was treated confidentially and reminded that refusal to participate was possible at any time. Written informed consent was then obtained. In total, 20 participants from Agequake part I (countries C1 (Switzerland), C2 and C3) and 63 from Agequake part II (countries C4 (Switzerland) and C5 (Canada)) were included in the sample for this paper. Details on the number and type of prisons included are reported elsewhere [20, 21]. (We cannot reveal the country for C2 and C3, as we do not have consent from participants to do so [22].) The included participants of the first part of the study are presented in the following Fig. 1.

**Fig. 2:**
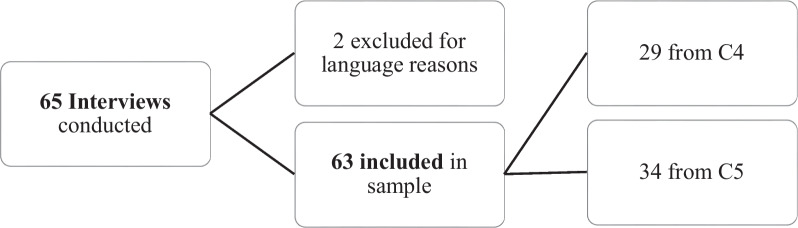
Participants recruitment and inclusion of Agequake part 2

The recruitment and inclusion of the second part of the study are presented in Fig. 2. At the time of data analysis, we decided to exclude the two participants from the Italian speaking part of Switzerland since they were not involved in providing mental health care.

In both parts, the interviews took place in German, English and French. First part interviews were carried out by PhD students and post-docs and by two PhD students in the second, all of them trained in qualitative interview techniques. The interviews were mostly one-on-one and face to face in a separate room; due to cost and time, the interviews with participants located abroad were conducted via Skype or telephone. Privacy was maintained to enable participants to talk freely. Upon consent of the participants the interviews were audio-recorded and subsequent transcribed verbatim. The transcripts were checked for quality and accuracy of the transcription, all identifying information were anonymized.

The questions in the interviews concerned the experts' experiences, including why they chose to work in prisons, perception of healthcare, and its accessibility for ageing incarcerated people (in their particular region or country) (see [10] for more details). For the mental health continuation of the study, they were also questioned about their work experience, and about organization of mental health care, role conflict issues and risk assessment.

In this work, we further analysed the interviews of the healthcare providers only since the research question for this study concerns their motivation to work in this context. The data was coded using Mayring’s methods of qualitative content analysis into themes responding to the study aim using qualitative analysis software MAXQDA2020. During our analysis, first, we looked at how experts decided to start working in prison. Thereafter we analysed how their training and motivation could have implications on their work and their perception of difficulties encountered in health care provision. We also evaluated experts' opinions about perceived future training needs for working in prisons and identified specific limitations described concerning their work in prison. We present our analysis under the following three themes: reasons for working in prison; perceived training needs; and limitations to ensuring quality of health care in prison.

## Results

The 20 participants included in the first part of the study from Switzerland (C1) and two other Western European countries (C2 and C3) belonged to the following healthcare professions: physicians (n = 6), psychiatrists (n = 4), nurses (n = 5), physical therapist (n = 1), pharmacist (n = 1), healthcare researchers (n = 2) and prison health inspector (n = 1). The 29 participants from Switzerland (C4) in the second part of the study belonged to the following healthcare professions: psychiatrists (n = 17), psychologists (n = 6), nurses (n = 6). Finally, the 34 interviews conducted in Canada (C5) included in the second part of the study belonged to the following healthcare professions: psychiatrists or psychologists (n = 12), nurses (n = 4), managers or mental health team leaders (n = 12), social workers (n = 2), researchers (n = 3), legal coordinator (n = 1). The professional backgrounds of the participants included in this sample are summarized and presented in Table 2.

**Table 2:** Participants background of health care professionals included in the sample

	Somatic care physicians	Psychiatrists and psychologists	Nurses	Other professions*
Agequake first part	6	4	5	5
Agequake second part C4 (Switzerland)	0	23	6	0
Agequake second part C5 (Canada)	0	12	4	18
Total	N = 6	N = 39	N = 15	N = 23

*Physical therapist, pharmacist, health inspector, managers, team leaders, social workers, legal coordinator, researchers

## Reasons for working in prison

### Practical reasons

Each participant had individual reasons for working in a prison environment. Nevertheless, there was some overlap in their motivations. For some interviewees it was coincidence to find an employment in the prison, as the institution was in an understaffed condition.

P108 C4, psychiatrist:* This happened by coincidence because… where I worked back then, the assessment field was understaffed […]

P111 C4, psychiatrist: I have to say that I ended up in the forensic psychiatry more or less by coincidence.

Previous experiences in prisons, whether as interns or as trainees, influenced participants by making them consider working in a prison environment. Two interviewees mentioned early contact during education as reasons for working in this setting. Two more interviewees had early contact with the subject during university. Another participant started to work in correctional institutions for a clerkship and chose a career in this setting.

P12 C3, general practitioner: [No it isn’t my first experience with detainees], I did a six month internship when I was a general medicine resident.

P228 C5, chief mental health services: […] uh When I was doing my doctorate so we’re talking about 25 years ago about uh I chose as a thesis subject to work with uh psychopathy so it was already a subject of interest to me at the time of my studies.-

P231 C5, psychologist: Uhm, actually I started an entry uh uh level job in corrections as a clerk uhm […] and it was my intention to actually stay uh with corrections as a career and and I have done so to date.

More forms of early contact were mentioned as influencing interest in correctional institutions and generating motivation to work there. One participant named the environment he grew up in as the reason for his interest in the population he is now working with. Knowing the environment of his patients improved his relations with them:

P236 C5, psychologist: Well my motivation to work in corrections uh is primarily because of my background growing up/ I grew up in (City area in Canada) […] and there are a lot of gangs and there is a lot of prostitutes and uh I grew up with […] all my life you know. Like uh we were in uh relatively poor family so I found that I could relate to these people really well.

A few experts found themselves working in prisons mainly for structural reasons. These reasons were beyond their control and included an institutional modification or a specific relation between the expert’s home hospital and a prison. For instance, three participants reported working in prison since their work setting was affiliated with a prison:

P6 C3, emergency physician: So, I have been trained as [an]emergency physician in the university hospital of [name city], where the prisoner unit is attached to the emergency department […].

P24 C1, forensic psychiatrist: The prison [name] has a partnership with the clinic [name] and I am a senior physician in psychiatry. I have been working in the forensic field for a long time …. It is an independent task, a service for [prison name] which consists in ensuring the patients’ healthcare.

P123 C4, nurse**:** Previously I was instructor, well I was professor in a college of nurse trainees in [Name of City in C1] and I had a reference or a internship in a prison.

One participant experienced a change in the institution itself that led to an employment in the forensic field. Likewise, a mental health nurse reported that she was transferred from hospital to prison due to institutional changes.

P130/131 C4, nurses: So I did not have much of a choice because for my function as a superior nurse while the institution and the mandate of public-health opened a nurse service for prison environment and well I found myself naturally creating/ establishing a team of nurses for ensuring nursing in prison environment.

P35 C2, mental health nurse: And then for eight years, I was a general manager for medical services … prior to becoming head of prison health care.

For some, the contact with penal institutions developed through private contacts such as friends or relatives. The private access reduced hesitation to work in a prison environment.

P25 C1, general nurse: I already knew a little bit about [this work] through a former acquaintance of mine. I had therefore a certain understanding of how the system functioned. This is why I didn’t need to over think it too long as I saw the job advertising.

### Intrinsic reasons

Even though many participants brought up more coincidental reasons, many decided to work in a prison environment on a basis of inner conviction and interest. The different intrinsic drive varied among the participants, but some general tendency in the motivations was detectable. We divided these into three subthemes.

#### Public impact as a therapist in a prison setting

The motivation was a hope to find a way to change the correctional system. Two participants wanted to have a direct impact on people living in detention because they were unhappy with how the justice system was managed.

P207 C5, occupational therapist: And they were uh/ they used a lot of black humor um to try to cope. […]it just really shocked me that these are people that come to us for help um and this is how treat them and this is how we talk about them. … Um but it really motivated me to start uh trying to see if I could get inside and do a, you know do a system change, at least within an organization um in the way people are seen.

P219 C5, research scientist: Um so uh I have frustrations with our criminal justice system. […] And so um I wanted to work/ I felt like I had the most power to influence individuals rather than the system. […]Um so I wanted to work in corrections originally. Um but just sort of by chance ended up at a psychiatric hospital um on a clinical unit.

Crime prevention was often mentioned as motivation. The hope to change the inmates lives after prison for the good and therewith prevent forthcoming crimes for some was cause for a start to work in prison. Some even felt a public responsibility to ensure public security.

P125 C4, psychologist: For me personally, the motivation is actually the child’s protection or the victim’s protection. […] And this is for me motivationally the main aspect. Aside [from] the social rehabilitation intention and aside [from] the victim protection intention [intention to protect the victim] also a good future for the affected.

127 C4, psychologist: The motivation always was the interest to it, […] so my interest always was there for ehm analytics of crimes and also of criminals and what someone leads to or what causes someone to commit such an act and how can one make someone to stop doing it and in general how can one prevent people to do such things, so with prevention strategies[…].

120 C4, psychologist: […] and then there is the side of recurrence prevention that I’m interested in, in the sense of you have a real, ehm, with this act you have a real impact in public security. Ehm, so we have responsibilities that come with the function that I find interesting.

#### Possibilities as a therapist working in prison

Another reason often mentioned by participants was the possibilities that one has as a therapist in a prison setting. In regard of the fact that the patients in prison have restricted access to medical staff, the therapists have to treat every aspect (“the whole patient”), meaning the mental health issue and the physical issues.

P224 C5, occupational therapist: And you know, as an occupational therapist in mental health you are really treating the whole person, whereas if you specialize in physical health sometimes you feel like you are treating like one part of the body almost. Or uhm so I feel like going in that direction with my career.

For other participants, the motivation to work in prison was to give detained persons and in particular elder persons a dignified and thorough treatment, to give them their full attention. The observation was made, that the elder the detainees were, the harder it was to find reasonable solutions.

P124 C4, psychiatrist: Ehm to take care, that someone is active for the accused, for the explored, who is working thorough, this is important for me and to take the person seriously, as hard as it is, to try to recommend rational solutions. […] But again the motivation from my side, to provide the service is obviously to do this as thoroughly as possible and to completely get involved with the topic.

Some participants said that their reason to work in a prison was their interest in the interface of law/psychiatry or law/medicine that working in correctional institutions offered.

P126 C4, psychiatrist: I have, by the time I didn’t know what to study exactly, thought about liking law. Nevertheless I decided for medicine (laughs) and after that I actually found myself a way to combine the two things.

P201 C5, psychologist: It has been some time now (both laugh). Um it is just area of interest. How it combines care/ mental health care and the legal aspect of it as well. Very interesting.

#### Natural interest in prison population and their biographies

Other participants mentioned their basic interest in the people’s lives and coping strategies for living with substantial burdens; by working as therapists and by writing consultations they could satisfy their curiosity.

P121 C4, psychologist: and one area of my interest was already with marginalised people, people on the edge of society, in difficult situations. […] How [do they] manage and how do people live in such a situation? With the burden they have put on themselves with their history, with the crimes they have fallen into.

P221 C5, social worker: I really liked the idea of working in an institution that/ get to see kind of a wide range of, you know, social issues, mental health issues, that are reflected in our community as well. So I just really kind of come here and work in an environment that gave me exposure to all of that.

P230 C5, clinical team leader: I was interested in working with an older population, interested in addiction, interested in trauma and it all kind of comes together in the prison environment.

## Perceived training needs

Healthcare professionals working in prison received training in different disciplines of medicine and nursing such as internal medicine, sports medicine, or psychiatry. The range of specialisation ranges from no further training after nursing school (except working experience), to specialisation with a certificate of advanced studies.

P106 C4, nurse: ok uh I've been working in this field for 15 years, so in the medical department of prison psychiatry and then in fact uh I did my last internship in nursing school at the time it was a 3 month internship uh in the [name of prison] in [name of city], a prison where there are women and men.

P227 C5, nurse: In fact, what they require is a minimum of two years' experience as a nurse. And then I, as I was in mental health, of course it was also a requirement to have two years' experience in psychiatry.

P111 C4, psychiatrist: no in general psychiatry I don't have a specialisation in prison psychology […] so at the moment I'm doing the Certificate of Advanced Studies in forensic psychiatry and forensic psychology […] in medical-legal psychiatry, yes, because I was also doing expertises [expert opinions]

P120 C4, psychologist: I worked as a clinical psychologist, I did assessment, I worked in neuropsychology, and then I trained …. in forensic psychology, and then I did a specialist title in forensic psychology.

Irrespective of this diversity in professional backgrounds, some participants reported being sufficiently trained to work in this setting while others saw a need for further education. For instance, one nurse thought that general nurse training was sufficient for working in prison, as long as it was possible to contact the hospital if required.

P8 C3, nurse: For me, [this work] resembles what I was doing before. It is similar for nurses. Basically, we have a training and if ever I need an answer in a specific field, […] we have ways to get this answer. At the hospital level we can get all the necessary resources. […] I do not need [additional] training at all and I believe that nurses do not have the need either.

However, a general problem that was often mentioned was the broadness of the population. To offer appropriate medical care, it often was not enough to have a general medicine specialisation, as the complexity of the patients demanded specialisations in all possible fields. This problem was found in various forms, e.g. psychiatrists with a lack of general medicine knowledge or general practitioners with very limited geriatric specialisation. Further examples were reported from nursing, such as nurses who are trained in general acute medicine and lack of know-how for nursing care of sex offenders, for example.

P217 C5, occupational therapist/unit manager: Because right now we're becoming a little bit of a catch-all and um the benefit of that is that we have lots of resources so we can kind of uh provide uh individual services. But the difficulty of that is having staff trained in everything … you can't be a specialist - you can't be a general specialist right?

P6 C3, emergency physician: I had never practiced general medicine but my two colleagues had gained a little experience in the field a few years ago. I had only done my emergency medicine training and I went directly to the hospital. So it was a bit complicated for me in the beginning […].

P12 C3, general practitioner: I did not feel properly trained [in geriatrics], at least from a medical point of view, because before that I used to apply my knowledge in adult medicine to the elderly although they belong to a very specific population. […]

Many professionals reported the problem of providing care for geriatric patients, on the one hand in form of treating typical geriatic illnesses and on the other hand in form of accompanying and caring for geriatic patients that face the end of their lives. Staff were often not trained enough for handling these cases.

P217 C5, occupational therapist/unit manager: Um you know because our staff isn’t necessarily trained to provide care to individuals with dementia. […] there's an old/ elderly gentleman who's been deemed palliative care. […] we don't do the things necessarily that um other programs may do much more naturally because they’re used to working with clients who are elderly. Um so you know [a] palliative care consult hadn’t been done again.

P102 C4, psychiatrist: The formation is designed for a young/ um for a young adult population[…]Where they have difficulties is with very young patients, as well as with very old patients… because special questions arise there. But someone who has a good basic training should, from my point of view, be able to cover that as well.

Another participant described as problematic the education of new physician trainees in the prisons, which was useful to train young doctors in prison medicine but consumed time and meant doctors lacked experience.

P25 C1, nurse: […] Here are working the trainees in internal medicine who are doing a rotation. So, they are in different services in [name of hospital] and they normally have to work half a year [in prison]. […]This system is sometimes expensive, because each time they must be taught from the start and it takes a while before they understand how it functions here and before they know a bit more what to do. […]

Concerning the recruitment of young physicians, the public and media-derived picture of working in prisons was mentioned to be a reason why there are too few new recruits.

P103 C4, psychiatrist: Right now there are few people who are interested in this and particularly for expertises, because this scares [people] quite a lot. Because there is a public media exposition which is a responsibility that is very heavy and we see few young colleagues that are interested, finally it is difficult and frustrating, the expertises, in a certain way, so there are, we have trouble to attract.

Finally, one psychiatrist reported that dangerousness assessment was not taught in universities, although he considered this rather difficult task of the highest importance in his work in the prison context.

P34 C3, psychiatrist: […] Prison physicians who do not have the competences [to assess dangerousness] cannot be experts […].

## Discussion

The present study is important, timely and to our knowledge the first to provide data on motivation of prison physicians, perceived difficulties and training needs. From the literature to date it is evident that prisons lack qualified physicians and that there are practical barriers to accessing health care not only inside prisons, but also access to specialist care that might require transport to facilities outside the prison walls. This can lead to insufficient health care for patients in prisons and be the cause of reported negative experiences [11, 18].

Our results indicate that the reasons for choosing to work in prison varied. For most professionals the choice of working in prison had been facilitated by direct or indirect exposure to the prison environment. Various forms of early contact were reported, with some participants reporting first contact during their training (in medicine or nursing training), some during doctoral studies at university and some much earlier in their childhood, and most of the interviewees found this work interesting or fascinating, even though they had little knowledge of this complex setting that they often perceived as “frightening” at the beginning of their working career in prison. The work that health care providers perform in prison may benefit from a wider acknowledgment in the medical community since prison medicine is not the first or most obvious choice for a medical career for many young trainees. Moreover, early exposure to prisons could help young trainees to understand and appreciate health care in prison, and even develop their interest in a future career in this field [17].

With regard to the fact that many professionals also reported a first exposure to the prison environment due to structural reasons (e.g. emergency departments collaborating with prison units, psychiatry-prison partnerships) more health care professionals may consider a career in this field if they are able to do a rotation in a prison during their education and training. It may be enough to arrange a one-month internship for medicine students during the end of medical school to attract more professionals. Furthermore, our data show that the motivation to work in a prison environment can also grow out of discontent with how the justice system is implemented. Various interviewees mentioned their hope of changing the system by working there. To prepare health care professionals adequately it may be helpful to thematize the ethical questions which one faces while working with incarcerated patients, during study time or even—as a form of general public education—in high school. It also may be inspiring for students to see and hear the possibilities one has when working in prison to change an unjust system by getting to know professionals who work in prisons. This means of active confrontation may also help to correct the public picture of the prison working environment. As one participant mentioned, media often depict prisons as frightening workplaces that are burdened with exaggerated risks. This keeps young doctors and nurses from starting a specialization in this field.

Related to our above recommendation, our study findings underline that participants’ evaluation of whether their training and previous education was sufficient varied depending on the participants’ professions. On the one hand, the nurses who took part in this study seemed quite satisfied with their educational training. As long as the availability of medical staff and specialists was guaranteed, the nurses felt secure and sufficiently trained to handle the various somatic problems of the patients. Nevertheless, it was mentioned, that when it comes to taking care of patients with mental health problems, nurses often felt often overwhelmed. This was mostly attributed to insufficient training in mental health nursing. On the other hand, it seemed that as long as physicians received broad enough training, they saw themselves as well prepared to work in prison. However, the interviewed physicians reported a general lack of access to medical specialists in prison. Given the great variety of health problems among imprisoned patient populations, the access to specialists in every field is essential for appropriate healthcare. In particular, participants regretted the lack of specialists with adequate training in geriatrics. Given the increase of older patients in prison, experience in providing care for the chronic illnesses typical of older populations is essential and perceived as a critical problem of healthcare in prison. Better training in geriatric care as well as in end-of-life care is necessary, as it is evident that the ageing prison population has a higher prevalence in chronic diseases and more and more patients ultimately die in prison [11, 13, 18]. Furthermore, existing studies emphasize the need for ethical and legal training for physicians working in this context [23, 24]. Finally, concerning specific educational needs, one important point mentioned was the assessment of dangerousness, for which prison psychiatrists and other health care professionals report receiving inadequate training. This is of concern since health care professionals, in particular psychiatrists, could be asked by the judiciary system to provide an assessment for an incarcerated person [23–26]. One study concluded that forensic psychiatric assessment (at least for schizophrenic patients) may be subject to significant differences between psychiatrists, and suggest that appropriate training may improve this issue [27]. According to the view of the Swiss Federal Court, treating physicians and psychiatrists cannot be expert witnesses due to their conflicting obligations and specifically the partiality resulting from their professional obligation to put the benefit of their patients first (decision 6B_648/2014) [28]. However, this is not the case in other countries and even in Switzerland, after the sentencing, in court-ordered therapies the therapist is asked to take on a dual role and to also draft dangerousness assessments. Education in forensic matters is scarce in most medical schools and training is generally provided during postgraduate work by professional associations of forensic psychiatry and psychology. For example, the Swiss Society of Forensic Psychiatry provides training for physicians and psychiatrists who want to become forensic experts. The differences between jurisdictions not only in different countries, but also between regions such as states, provinces and cantons, increase difficulties in providing locally adapted training.

## Limitations

This study has several limitations. First and foremost, this paper analysed data collected as part of a project on ageing population in prison, and the goal of the overall project was not to understand how professionals chose to work in this setting. Thus, we can neither assume that the data was complete nor data saturation was reached for this particular topic as this was not specially assessed during the project. However, data saturation was reached for the key research questions that we sought to uncover for both the parts of the project. Second, our results do not claim to be representative of all prison healthcare practitioners' opinions or of the represented countries’ prison systems. Third, only experts who agreed to participate were interviewed. As this group can be considered a motivated one, there could be bias in their reporting. Fourth, the interviews were conducted by several interviewers who contacted experts by three different means (phone, Skype, and in person interviews). This might have resulted in some differences since in qualitative research, the interviewer is the tool of data collection. Finally, participants were not re-contacted to give feedback on the results to further validate the study findings.

## Conclusion

To our knowledge, there is no specific formally required training program related to work in prison healthcare services in Switzerland (as well as in other countries). A prison is a particular environment where healthcare providers may face distinct challenges. Physicians working in somatic fields, psychologists, psychiatrists and nurses would benefit from an early exposure to the prison environment [29, 30], or at least from additional training in prison medicine [6] in order to obtain a more realistic view of prison medicine and public health needs in this field. Prison health professionals would profit from continued education and training geared towards addressing the needs of an ageing population with multiple chronic conditions [7, 13, 31]. Training in legal and ethical issues related to healthcare in prison, including the right to health, the principle of equivalence of care and confidentiality would also be valuable to them. These trainings are important since physicians and nurses bear the main responsibilities for medical treatments and face precarious situations in addressing the needs of their patients in prison. The question remains as to whether all medical students would benefit from a first exposure to prison medicine during their medical education, which is the case in the canton of Geneva [2] or whether prison medicine could be sufficiently taught as a post-graduate program for future prison physicians [32]. One study indicates that a post-graduate program has been appreciated by interns [7]. Another solution could be to ensure that prison health care professionals have access to continuing education based on their individual needs either via general medical societies or via professional networks like the Conference of Swiss Prison Health Physicians, which also incorporates other health professionals such as nurses. National and regional medical societies should include prison specific training modules in their curriculum. Lastly, this study points out the need for more research to improve training and recruitment for prison health professionals to ensure that they are knowledgeable about legal and ethical concerns related to health care in prison and hence are able to uphold the principle of equivalence of care.

## Data Availability

The dataset used during the current study are not publicly available. We did not seek consent to publish entire interview transcripts due to the risk of breaching participant confidentiality and maintaining participant anonymity. Data are partially available from the corresponding author on reasonable request.
